# Corticosteroids as a conservative treatment for recurrent pediatric oral pyogenic granuloma: a case report and review of the literature

**DOI:** 10.11604/pamj.2024.49.51.44964

**Published:** 2024-10-23

**Authors:** Dorian Henry, Guillaume Gautier, Benjamin Faudemer, Julien Hamon

**Affiliations:** 1 Department of Oral and Maxillofacial Surgery, University Hospital of Caen, Caen, France,; 2 Department of Oral Surgery, University Hospital of Rennes, Rennes, France

**Keywords:** Pyogenic granuloma, steroids, pediatric, oral surgery, case report

## Abstract

Pyogenic granuloma is a noncancerous inflammatory tumor of the skin or mucous membranes. The most frequent causes include hormonal changes, drugs, or localized chronic inflammation. We report the case of an 11-year-old girl who presented with a bleeding gingival swelling and no significant medical history. The pyogenic granuloma presented as a sessile, lobulated, and violaceous lesion with fibrinous ulcerations and telangiectasias. The tumor recurred despite six surgical excisions. In this case report, we treated the latest recurrence with topical corticosteroids and triamcinolone injections, resulting in complete remission with no side effects. This case emphasizes the necessity for diagnostic certainty and the potential of corticosteroids as a less invasive and effective treatment for recurring pyogenic granuloma.

## Introduction

Pyogenic granuloma (PG), often referred as botryomycoma or lobular capillary hemangioma, is a non-neoplastic inflammatory tumor that develops on the skin or mucous membranes. The etiology is still unclear, but several factors such as chronic local inflammation, medication intake, or hormonal changes are implicated [1-3]. Pyogenic granuloma may appear at any age, from 4.5 to 93 years; however, it most commonly affects younger females and children [1,2]. Pyogenic granuloma presents as a soft lobulated lesion, either pedunculated or sessile, with a reddish to purplish color and telangiectasias. They are often bleeding and ulcerated. Its diameter ranges from some millimeters to several centimeters [1]. Intra-oral locations account for 75% of cases, with a higher prevalence in the maxilla than the mandible, and more frequently in keratinized gingiva [1]. It can also be found on the tongue, lips, palate, or peri-implant areas [1,2]. The treatment of choice is complete surgical excision of the lesion. Despite optimal management, the recurrence rate after excision is estimated at 15% [1,2]. Recently, other surgical procedures such as laser, cryosurgery, or medication treatments like beta-blockers have been considered. This report presents the successful treatment of a recurrent PG with corticosteroid injections in a child.

## Patient and observation

**Patient and observation:** an 11-year-old African girl was referred to our oral surgery department with a bleeding gingival swelling that had been gradually developing for 3 months. The patient was receiving orthodontic treatment, was not taking any medications, and had no medical or surgical history. There was no history of smoking or primary trauma. Her oral hygiene was excellent, and there were no clinical or radiographic signs of a dental infection.

**Figure 1:**
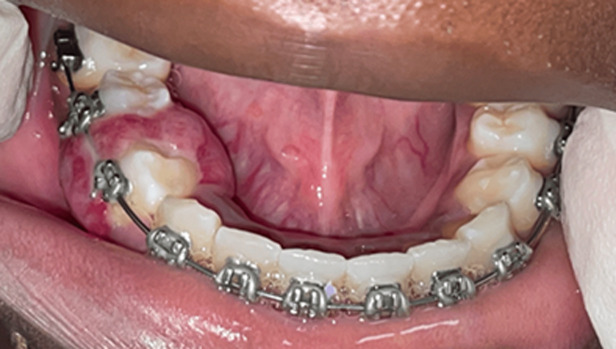
violaceous and lobulated lesion, associated with telangiectasias and areas of fibrinous ulcerations between teeth 44 and 45

**Clinical findings:** the lesion was violaceous, sessile, lobulated, and depressible, associated with telangiectasias and areas of fibrinous ulcerations. It measured 15x10x13mm and was hemorrhagic upon palpation. It was located on the gingival papilla between the mandibular teeth 44 and 45 (Figure 1).

**Timeline of current episode:** February 2019: first excision. February 2020: second excision. March 2021: third excision. June 2021: fourth excision. September 2022: fifth excision. October 2022: sixth excision. December 2022: first corticosteroid injection. January 2023: second corticosteroid injection. June 2024: last clinical follow-up consultation.

**Figure 2:**
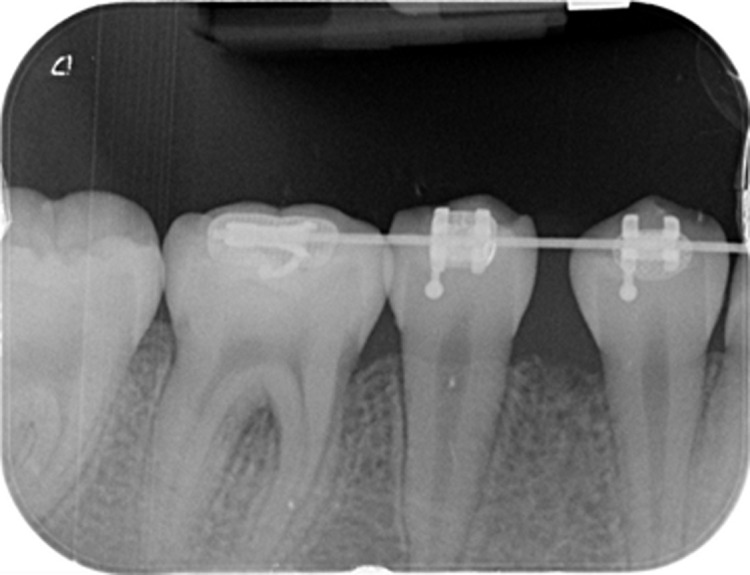
retroalveolar X-ray of teeth 44 to 47

**Diagnostic evaluation:** blood testing, including a complete blood count, returned normal. No anomalies of the adjacent teeth or alveolar bone were seen on the periapical radiograph or Cone-beam CT scan (Figure 2). Under local anesthetic, the lesion was removed with healthy margins. The resected specimen was preserved in a 4% formaldehyde solution, stained with hematoxylin and eosin (H&E), and embedded in a paraffin block for histological analysis.

**Figure 3:**
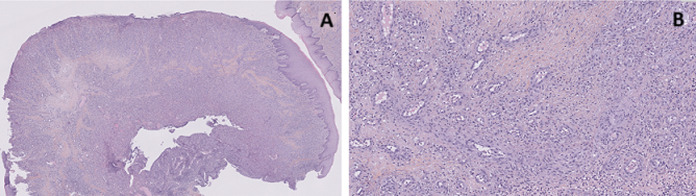
biopsy of the gingiva between the teeth 44 and 45; A) photomicrograph displaying hyperplastic surface epithelium accompanied by vascular proliferation H&E x5; B) photomicrograph displaying proliferation of thin-walled vascular structures lined by endothelial cells, grouped in lobules, and a polymorphous inflammatory infiltrate rich in neutrophils H&E x20

**Diagnosis:** histological analysis favored a benign vascular proliferation, suggesting PG. The malpighian mucosa, which was focally ulcerated, was raised by a lesion located in the chorion. The lesion was composed of numerous thin-walled vascular structures bordered with endothelial cells. The vessels were arranged in lobules, with a polymorphous inflammatory infiltrate rich in neutrophils (Figure 3).

**Therapeutic interventions:** the orthodontist removed the brackets from teeth 44 to 46, eliminating the secondary irritating factor. The patient also underwent debridements in the periodontology department. Despite six recurrences with repeated complete excisions, a multidisciplinary consultation decided to treat a new recurrence with delayed-release corticosteroid injections and topical corticosteroid applications. Antibiotics were given for one week to lower the bacterial load. After perilesional anesthesia with 0.6 ml of 1: 200000 adrenaline articaine, a 10 mg/ml triamcinolone acetonide (Kenacort Retard®) solution was injected intralesionally at five sites, with 0.1 ml at each. The patient was told to apply a 0.05% betamethasone ointment twice daily.

**Figure 4:**
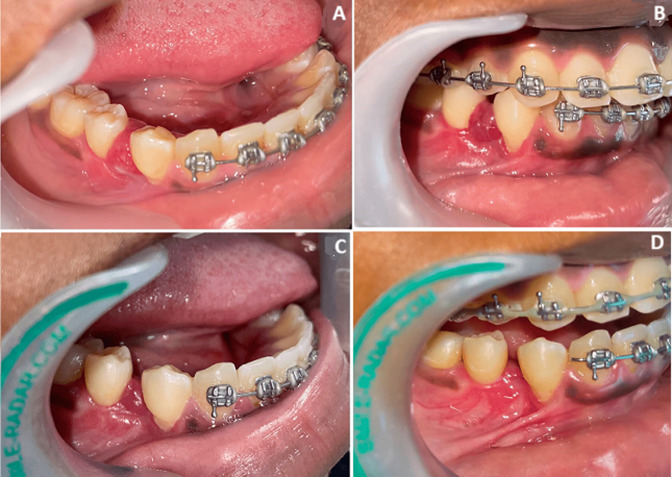
photographs of PG between teeth 44 and 45: A) before treatment; B) 2 weeks after first injection; C) 3 weeks after first injection; D) 2 weeks after second injection

**Figure 5:**
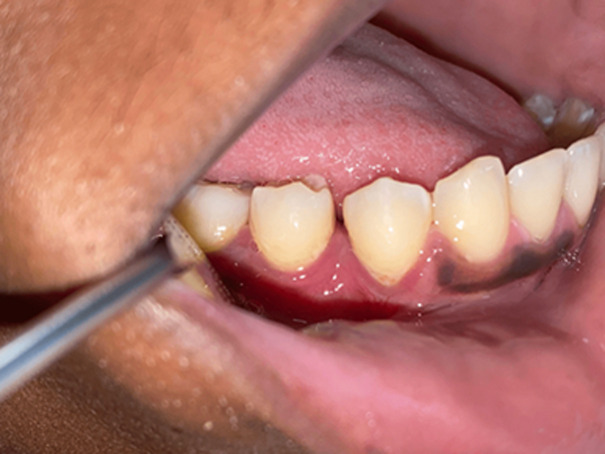
photograph of teeth 44 and 45, after 18 months

**Follow-up and outcome of interventions:** the postoperative course was uneventful, and no side effects were encountered. After two weeks, a reduction in inflammation and bleeding was observed. A second injection was performed according to the same protocol. Three weeks later, complete disappearance of the lesion and symptoms was observed, and the corticosteroid ointment was discontinued (Figure 4). The orthodontic treatment was resumed and completed. The patient attended follow-up appointments, and no recurrence was seen after 18 months (Figure 5).

**Patient perspective:** the patient experienced chewing pain that prevented her from eating properly. She and her parents expressed fatigue with the recurrent excisions. The proposed treatment was considered as remarkable because it was effective and minimally invasive compared to previous treatments.

**Informed consent:** written informed consent was obtained from the patient and her parents for diagnosis, treatment, and the use of images and clinical details for publication purposes.

## Discussion

This clinical case is original as it describes for the first time the treatment of a recurrent gingival PG with injectable corticosteroids in a child. Traditionally, its treatment requires complete surgical excision. Gingival locations, which are more likely to recur, should benefit from a 2mm extension in healthy margins, extended to adjacent teeth, removing the periosteum and accompanied by infra- and supra-sulcular debridement [1]. Other surgical procedures, such as sclerotherapy, which carries a necrotic and allergenic risk, cryotherapy, and more recently, Er: Yag or CO_2_ laser excision, show interesting results on bleeding and postoperative pain [1-4]. Pyogenic granuloma has an inflammatory and neovascular component, leading authors to draw an analogy with infantile hemangiomatous lesions. They observe an increase in proangiogenic factors, such as vascular endothelial growth factor (VEGF) and fibroblast growth factor (FGF), and a decrease in antiangiogenic factors, such as angiostatin [5]. In 2014, Wine Lee *et al*. proposed the use of beta2-adrenergic receptor antagonists in gels as a treatment for cutaneous pyogenic granulomas, assuming that this treatment, proven effective for infantile hemangioma, would also be beneficial for other acquired vascular tumors [6]. Mashiah *et al*. found that 72% of patients achieve complete or partial remission after an average of 6.5 weeks of treatment with 5% Propranolol [7]. However, the use of beta-blockers for oral PG is still not proven and there is no suitable galenic form on the market. Corticosteroids in local application, intralesional injection, or systemic administration have well-known anti-angiogenic effects by reducing VEGF, basic fibroblast growth factor (BFGF), and macrophage activity, and by decreasing phospholipase A2, a precursor of proangiogenic prostaglandins [8]. The use of corticosteroids is validated in the treatment of cutaneous PG, with injections in two patients and systemic administration in another [9,10].

Only two case reports focus on the use of corticosteroids in the treatment of intra-oral PG. In 2006, Parisi *et al*. described the treatment of a recurrent oral PG with intranodular triamcinolone injections in a 33-year-old patient, resulting in complete tumor disappearance [3]. In 2015, Bugshan *et al*. used a highly concentrated triamcinolone acetonide solution to treat a 51-year-old patient, resulting in total lesion elimination with no adverse effects or recurrence [2]. In our case report, we adhere to the authors' defined protocol. The treatment was effective from the first injection, reducing bleeding. After the second injection, the PG completely disappeared, and no secondary excision was performed. The initial experience was very difficult for our young patient due to the high number of recurrences, probably associated with difficult and incomplete excisions. Corticosteroid therapy was perceived as remarkable, being very well tolerated by the patient and causing no side effects. It is essential to note that this treatment should be considered only after a histological test has confirmed the diagnosis. Vascular tumors such as hemangiomas, oral fibromas, peripheral giant cell granulomas, peripheral ossifying fibromas, peripheral odontogenic fibromas, and neoplastic lesions including metastatic carcinoma, non-Hodgkin's lymphoma, and Kaposi's sarcoma are among the differential diagnoses to rule out [1]. Corticosteroid side effects must be closely monitored both locally and systemically, particularly in growing children. Well-conducted studies are necessary to confirm this approach and determine the minimal dosing required for PG disappearance.

## Conclusion

This case report reveals that corticosteroid injections can be an effective and less intrusive treatment for recurrent gingival pyogenic granuloma in children. In addition to being well tolerated by the patient, the treatment completely cured the lesion and avoided the need for additional surgery. These outcomes show that corticosteroids might be an effective alternative to recurrent excisions, given that the diagnosis is clearly established and the patient is monitored to control any possible side effects. This approach underscores the importance of exploring conservative treatments in pediatric patients with recurrent oral lesions.
